# Flash-Based Computing-in-Memory Architecture to Implement High-Precision Sparse Coding

**DOI:** 10.3390/mi14122190

**Published:** 2023-11-30

**Authors:** Yueran Qi, Yang Feng, Hai Wang, Chengcheng Wang, Maoying Bai, Jing Liu, Xuepeng Zhan, Jixuan Wu, Qianwen Wang, Jiezhi Chen

**Affiliations:** 1 School of Information Science and Engineering, Shandong University, Qingdao 266237, China; 202132729@mail.sdu.edu.cn (Y.Q.); feng.yang@mail.sdu.edu.cn (Y.F.); 201800272037@mail.sdu.edu.cn (H.W.); 202132732@mail.sdu.edu.cn (C.W.); maoy.bai@mail.sdu.edu.cn (M.B.); zhanxuepeng@sdu.edu.cn (X.Z.); jixuanwu@sdu.edu.cn (J.W.); qw.wang@qust.edu.cn (Q.W.); 2 Key Laboratory of Microelectronic Devices and Integrated Technology, Institute of Microelectronics of Chinese Academy of Sciences, Beijing 100029, China; liujing@ime.ac.cn

**Keywords:** computing in memory, sparse coding, image reconstruction, online training, flash memory

## Abstract

To address the concerns with power consumption and processing efficiency in big-size data processing, sparse coding in computing-in-memory (CIM) architectures is gaining much more attention. Here, a novel Flash-based CIM architecture is proposed to implement large-scale sparse coding, wherein various matrix weight training algorithms are verified. Then, with further optimizations of mapping methods and initialization conditions, the variation-sensitive training (VST) algorithm is designed to enhance the processing efficiency and accuracy of the applications of image reconstructions. Based on the comprehensive characterizations observed when considering the impacts of array variations, the experiment demonstrated that the trained dictionary could successfully reconstruct the images in a 55 nm flash memory array based on the proposed architecture, irrespective of current variations. The results indicate the feasibility of using Flash-based CIM architectures to implement high-precision sparse coding in a wide range of applications.

## 1. Introduction

Frequent data transportation between the memory unit and the computing unit causes huge power consumption and low processing efficiency. Computing-in-memory (CIM) technology was proposed to address the aforementioned issues and has been widely used in various applications, such as neural networks [1,2], scientific computing [3], image processing [4,5,6,7], etc. As an important part of image processing, the image reconstruction technique has been utilized in image super-resolution reconstruction [8], face recognition [9], and dynamic video anomaly detection [10]. Sparse coding is an unsupervised learning method that is usually used in machine learning because it is a powerful approach to high-efficient data representation [11,12,13,14,15,16,17], wherein the main goal is to find an overcomplete set of basis vectors to represent the input vector as a linear combination of basis vectors. In sparse coding, complex data can be transformed into a more simplified and meaningful form; however, the data size is ultra-large, and processing efficiency is a challenge. Fortunately, CIM-based sparse coding provides a promising solution to this.

So far, the relevant research has been implemented in memristor-based CIM architectures. Sheridan et al. [13] proposed the winner-take-all (WTA) method to train the dictionary on memristors, achieving image reconstruction via sparse coding, and then adopted the stochastic gradient descent (SGD) method for on-chip dictionary training [14]. By combining sparse coding and a single-layer perceptron (SLP) network, Cai et al. [15] demonstrated the use of an integrated memristor chip to recognize a breast tumor, checking malignancy or benignity; Kang et al. [16] proposed a cluster-type CBRAM device model to complete color image reconstruction; Dong et al. [17] proposed a training method (CP) with a threshold-type memristor model based on sparse coding in the super-resolution reconstruction of images. For images with high resolution, the data need to be stored and manipulated in a large memory array, which is a challenge for memristor-based CIMs. Additionally, the data have to be divided into small slices [18,19]. Flash memory demonstrates good performance in durability, speed, and cost-effectiveness, which provides a promising candidate for large-scale and high-precision computing. Previously, Flash-based CIMs have been reported in various applications [20,21,22], demonstrating their capabilities in large-scale data processing.

In this work, we propose an online algorithm based on a novel design of flash arrays to implement sparse coding and high-robust color image reconstruction. The major contributions of this work are as follows:

- A novel flash-based CIM array is proposed to implement forward and backward calculations. By treating the flash cells as resistors and connecting the inputs and outputs to the rows and columns, the forward and backward calculations are implemented in the same flash array, which is helpful in reducing the array area and improving processing efficiency.
- A new training method is proposed to reduce iteration numbers for low power consumption. The discrete cosine transform (DCT) dictionary was introduced in the initialization to train the overcomplete dictionary. According to the characterization results, it is shown that the new initialization method can improve training efficiency and reconstruction accuracy effectively.
- A variation-sensitive training (VST) algorithm is proposed to address array variations. Different training methods have different sensitivities to the degree of device variation, and the proposed VST method can achieve good reconstruction results by setting the training share of various training algorithms to the variation level.
- The mapping method is optimized to store the matrix with positive/negative weights. In comparison to the traditional method of storing positive/negative weights in a differential pair or separate arrays, the normalized mapping method enables array area reduction.

## 2. Materials and Methods

### 2.1. Flash Memory

The conventional von Neumann architecture cannot always meet the increasing demand for efficient data processing since the frequent data transmission between the memory unit and computational unit costs too much energy rather than computation. The emergence of computing-in-memory (CIM) technology can largely reduce power consumption and delay costs during data transmission. For certain computations, such as matrix and vector multiplication (MVM), the calculation process can be performed on the memory device [23,24]. Therefore, there is no need to transmit data to the computing unit to complete calculation tasks, saving a significant amount of energy. In particular, based on the current characteristics in the saturation region of flash, the drain current is independent of the drain-source voltage and determined solely by the gate voltage, which means that the flash cell can be considered a gate bias control variable resistance device. In MVM, each input vector element needs to be multiplied with the corresponding matrix element, and then, the products are summed to obtain the results, which can be easily realized via a single-read operation on a flash memory array. That is, all the matrix elements can be mapped into the flash array in the form of the threshold voltage (V_th_) according to specific mapping rules. With the voltage proportional to the input vector elements applied at the word line (WL), the charge per column of the array calculated by integrating the current at the bit line (BL) is the actual result of MVM. The entire process obeys Ohm’s law and Kirchhoff’s law.

### 2.2. Sparse Coding

Sparse coding is a technique for data compression and feature extraction that can reduce the data dimensionality and complexity by extracting the essential features and representing the data as a linear combination of features [25,26]. The whole process is similar to human brain computing, in which we only need to mobilize as few brain areas as possible to consume the least amount of energy to achieve the calculation task for a familiar knowledge point, and the calculation speed increases at the same time, which is the benefit of sparse coding [27]. Its mathematical meaning is to train a set of basis vectors $D=\left[d_{1},d_{2},\ldots ,d_{n}\right]\epsilon {\mathbb{R}}^{m\times n},n>m$, which can be considered a dictionary set; $d_{n}$ is the $n^{th}$ basis vector, called the $n^{th}$ feature dictionary in other words. As a result, all input data x can be represented as a linear combination of these basic dictionaries.

(1) x=Da,

where $a={\left[a_{1},a_{2},\cdots ,a_{m}\right]}^{T}\epsilon {\mathbb{R}}^{m}$ is the sparse set of coefficients, and only a small fraction of the elements is non-zero.

Then, to obtain the solution, the problem can be simplified and expressed as finding the optimal solution that minimizes the energy function if D is known as follows:

(2) $$\underset{a}{\min}\|x-Da{\|}_{2}^{2}+\lambda \|a{\|}_{1},$$

where x is the input signal, a is the neuronal activity coefficient, λ is the adjustment parameter, $\|\cdot {\|}_{1}$ denotes the L1 norm, and $\|\cdot {\|}_{2}$ denotes the L2 norm, governing the sparse representation error and sparsity, respectively. L1 norm and L2 norm are considered jointly to achieve the best representation of the input vector with fewer features.

To solve the problem above, the local competition algorithm (LCA) has been proven to be an efficient algorithm [28] and has shown its ability to solve sparse approximation problems on FPGA [25]. The basic idea of LCA is to divide the search space into several local regions, each region having its share of competing individuals who compete and cooperate to find the local optimal solution. While in the global search process, each local region competes and cooperates to find the optimal global solution. Its mathematical expression is shown as follows:

(3) $$\frac{du}{dt}=\frac{1}{\tau }\left(p-u-\left(D^{T}D-I\right)a\right),$$

(4) $$a=T\left(u,\lambda \right)=\left\{\begin{array}{c} u,u>\lambda \\ 0,u\leq \lambda \end{array}\right.$$

where u is the neuronal membrane potential, $p=x^{T}D$ is the projection of input signal x onto the dictionary, τ is the time parameter that controls the response rate of the neuron, and I is the unit matrix. The first term in Equation (3) can be regarded as the positive stimulus of the membrane potential, the −u term as the leakage term, and the $-\left(D^{T}D-I\right)a$ term as the lateral inhibition of other neurons. The value of $D^{T}D$ can measure the similarity of each neuron to ensure that similar neurons are not active at the same time via lateralizing inhibition. The coefficient a is defined according to the thresholding function defined in Equation (4).

Since the major operations in Equation (3) are based on MVM, as discussed above, it fits well with the design of the flash array. To avoid the matrix and matrix multiplication terms, $D^{T}D$, $x_{r}=Da^{T}$ is introduced instead; then, the whole formula can be converted into the following format with only addition, subtraction, and matrix-vector multiplication:

(5) $$\frac{du}{dt}=\frac{1}{\tau }\left(-u+{\left(x-x_{r}\right)}^{T}D+a\right),$$

### 2.3. Flash Memory Array Design

**Figure 1:**
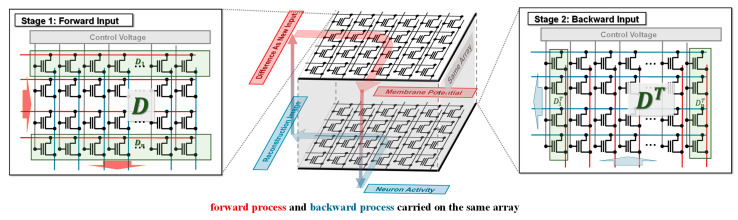
Schematic of the newly designed flash array for forward and backward input process. Forward calculation (red) can be implemented by applying the fixed amplitude voltage to the drain side and integrating the current at the source, while the back–forward calculation (blue) is the opposite. Each row or column (green) means a dictionary element of the dictionary set.

After eliminating the matrix and matrix multiplication terms that are not easily implemented in the array, the entire formula can be carried on the flash array. The dictionary D is mapped to the flash array and stored as Vth, and input x is mapped as the applied pulse, with the pulse time adjusted according to the input value at a fixed amplitude. Then, the computation of $x_{r}$ becomes the pivotal issue. In fact, a needs to be inputted into the array where the transpose of D is stored, so it requires two arrays to store the data of D and the transpose, respectively, which increases the device area. Since D is already stored in the array, we can implement this process by re-inputting a into the original output side, i.e., back input. However, the conventional flash memory array has difficulties in realizing back input, so we designed a new array where forward input and back forward input can be carried on in the same array to save the additional energy consumption of two arrays. The specific process is shown in Figure 1.

### 2.4. Grey Patch Reconstruction

A significant application of sparse coding is image processing. An image can be represented as a set of sparse coefficients that are full of information about the appearance features after the sparse coding process. These coefficients can be used for tasks such as image classification, target detection, and image reconstruction.

**Figure 2:**
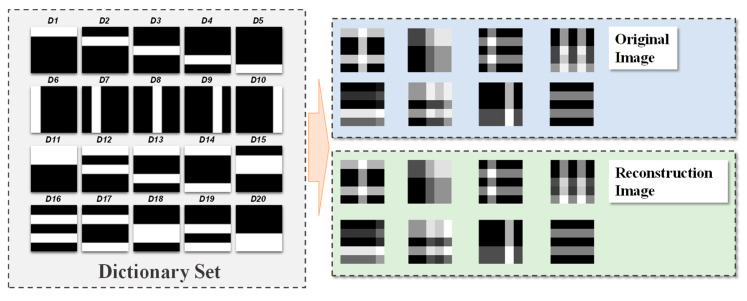
(Left): the given dictionary set. (Right): reconstruction results of small-size grayscale images.

To demonstrate the flash-based sparse coding system, an image reconstruction task is applied to the flash CIM array. Specifically, the LCA is implemented for validating the proposed CIM architecture. Using the commercial 55 nm flash memory technology, the reconstruction results of small-size images with black and white pixels are simulated. The dictionaries used during reconstructing images (Figure 2) were mapped to Vth of the array with the structure shown in Figure 1; then, the grayscale images to be reconstructed were converted to column vectors and applied to the array as fixed amplitude pulses with a duration proportional to the gray value. By iterating the forward and backward inputs, the results integrating at the output called “the membrane potential” will reach dynamic stability, at which time the dictionaries corresponding to the neurons whose potential is above the threshold will form the reconstructed image. The reconstruction results are shown in Figure 2, confirming the great performance of flash-based CIM architecture with “back input” operation.

**Figure 3:**
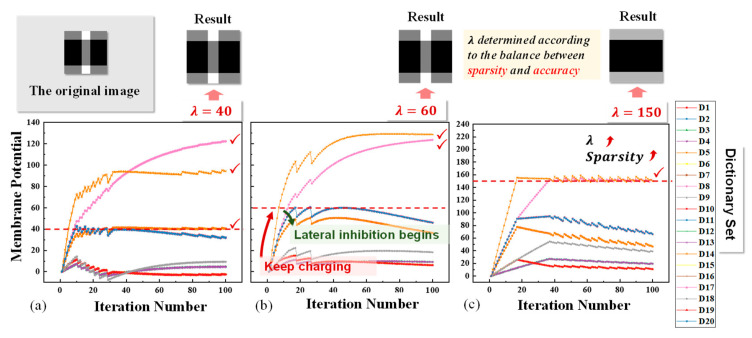
The trend diagram of membrane potential (**a**) when λ is 40, (**b**) when λ is 60, and (**c**) when λ is 150.

Then, to investigate the effect of the threshold parameter λ in LCA, we simulated the reconstruction results with various λ values. The membrane potential changes during the reconstruction are shown in Figure 3. It is observed clearly that when the membrane potential is below the threshold, the neurons corresponding to the dictionaries that reassemble the original image are charged rapidly until above the threshold. At the same time, the lateral inhibition is activated to reduce the membrane potential, and the correct neurons reach stability in the constant alternation of charging and inhibiting while the membrane potential of several irrelevant neurons starts to decrease. Also, it is noticeable that the value of λ affects the reconstruction outcome. As shown in Figure 3, neurons 1, 5, 8, 14 all remain active when λ is 40, only neurons 8 and 14 are selected when λ is 60, and only neuron 8 is selected when λ is 150, from which we can deduce that λ affects the number of active dictionary neurons, that is, sparsity in reconstruction.

### 2.5. Color Image Reconstruction

**Figure 4:**
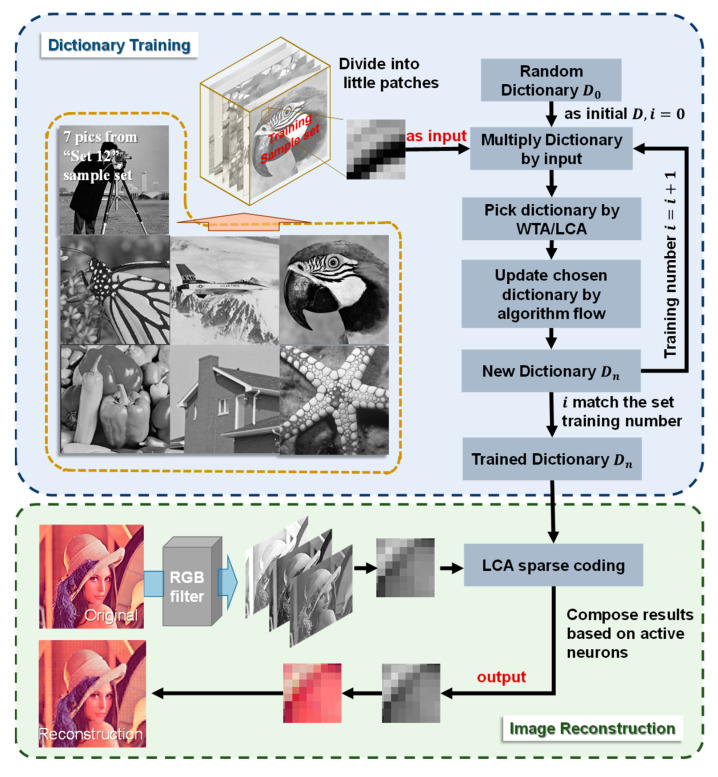
Schematic diagram of the architecture of image reconstruction.

The same architecture is used to implement color image reconstruction tasks with the entire process described in Figure 4. The first issue that needs to be addressed is the selection of dictionaries, which could greatly influence the reconstruction results. Once the basis dictionary is selected, all other inputs can be uniquely represented as linear combinations of dictionaries. The dictionary is usually trained using iterative algorithms that allow the dictionary elements to gradually adapt to the features of the input data. Various training methods have been proposed, and the comparative analysis of different dictionaries is performed on image reconstruction, focusing on three training algorithms: winner-take-all (WTA) algorithm, CP method [17], and stochastic gradient descent (SGD) algorithm.

#### 2.5.1. WTA

The winner-take-all (WTA) algorithm is an effective approach in selecting the most relevant features in input data and reducing the computational complexity of the neural network. This algorithm chooses the most active neuron amongst a layer of the network and inhibits all other neurons within that layer, resulting in the suppression of their activation levels. This process enables the network to quickly identify the most important features of the data, thereby discarding any irrelevant information. Therefore, the WTA algorithm improves the efficiency and accuracy of the neural network by prioritizing the significant features of the data. Therefore, instead of using the LCA algorithm to extract image features, the WTA algorithm directly updates the neurons that are most similar to the input image. The specific algorithm flow and the weight update equation can be found in [13].

However, one limitation of the WTA algorithm is that it can only update a single neuron with each input. This means that if there are multiple features that are equally likely, the algorithm may not be able to accurately identify the true feature.

#### 2.5.2. CP

This is an efficient training method proposed by [17], which we briefly call the CP method (the abbreviations ‘C’ and ‘P’ extracted from the training computation formula). Unlike WTA, this method requires the feature data extracted via LCA first, and then the weights are updated according to the formula in [17].

#### 2.5.3. SGD

The stochastic gradient descent (SGD) algorithm is a commonly used method in model training. As with the CP method, we need to extract features using LCA and then update neuron weights according to the SGD’s weight update formula in [13]. Specifically, the SGD algorithm only updates the weights of neurons that are active at the current input.

## 3. Results

In general, there are positive and negative trained dictionary weights, which are against the physical characteristics of flash memory devices. Previous work mostly deals with negative values by difference pairs, that is, using a pair of matrices, where one stores the positive part of the weight while the absolute value of the negative part of the weight is stored in the other one. Thus, the actual result can be obtained by subtracting the output of the two arrays. This method is feasible with increasing array area. To reduce the array area and power consumption, a new optimized mapping method is proposed to eliminate negative weights, reducing the array area as well.

(6) $$D_{n}=\frac{1}{\mu }\left(D-D_{min}\right),$$

where $D_{n}$ is the dictionary matrix after the mapping process, μ = $d_{max}-d_{min}$, $D_{min}$= $d_{min}\times \mathrm{ones}\left(\mathrm{size}\left(D\right)\right)$, $d_{max}$ and $d_{min}$ are the maximum and minimum values of the dictionary matrix, respectively, the $\mathrm{ones}\left(\mathrm{size}\left(D_{n}\right)\right)$ term means a matrix with the same size as D, and all elements in it are one.

Then, the actual result can be attained via

(7) $$y=\mu y_{n}+D_{min}x,$$

where $y_{n}$ is the output result, and the last term $D_{min}x$ can be obtained by summing over the input x.

**Figure 5:**
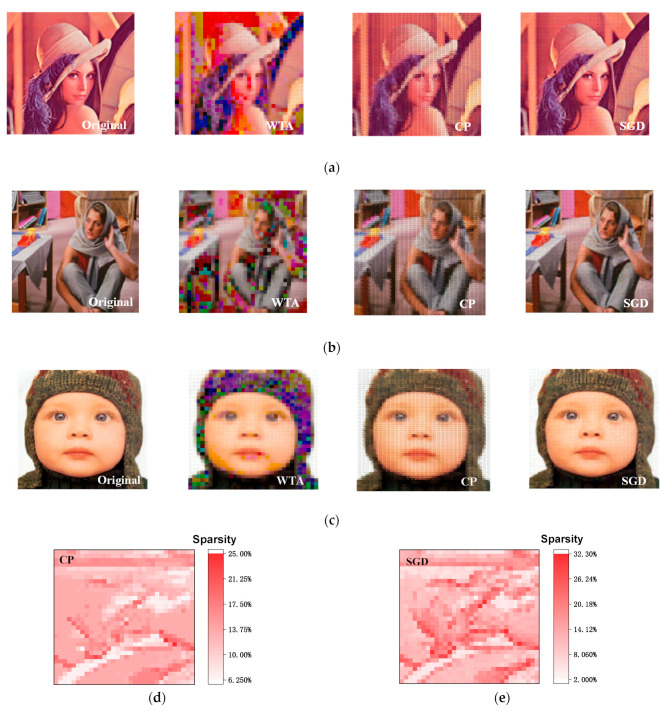
The reconstruction results of different image reconstruction targets: (**a**) Lena, (**b**) Barbara, and (**c**) Kid. From left to right, the original image and the results of the WTA method, CP method, and SGD method are shown, respectively. The sparsity of the Lena image reconstructions, i.e., the percentage of non-zero elements in a, with (**d**) CP and (**e**) SGD methods.

The array occupation caused by positive and negative matrices is solved, and the ability of flash arrays to implement online training has been proven [29]. Based on the above three algorithms, three online training methods were investigated; the reconstructed effect of different color pictures is illustrated in Figure 5 and Table 1. For comparison, we concentrated on three main parameters in the field of image processing: peak signal-to-noise ratio (*PSNR*), structural similarity (*SSIM*), and mean absolute error (*MAE*). These parameters are given via the following equations:

(8) $$\begin{array}{c} PSNR=10\times log\left(\frac{255^{2}}{MSE}\right),\\ MSE=\frac{1}{mn}\Sigma_{i=0}^{m-1}\Sigma_{j=0}^{n-1}{\left(O\left(i,j\right)-R\left(i,j\right)\right)}^{2} \end{array}$$

(9) $$SSIM\left(O,R\right)=\frac{\left(2\mu_{O}\mu_{R}+C_{1}\right)\left(2\sigma_{OR}+C_{2}\right)}{\left(\mu_{O}^{2}+\mu_{R}^{2}+C_{1}\right)\left(\sigma_{O}^{2}+\sigma_{R}^{2}+C_{2}\right)},$$

(10) $$MAE=\frac{1}{mn}\Sigma_{i=0}^{m-1}\Sigma_{j=0}^{n-1}\left|O\left(i,j\right)-R\left(i,j\right)\right|,$$

where $O\left(i,j\right)$ and $R\left(i,j\right)$ are pixel values of m×n sized original image and reconstruction image, respectively. μ is the average grayscale, and σ is the grayscale standard deviation.

Taking three different color images into consideration, the dictionaries trained with the SGD algorithm can all maintain good reconstruction results, while the results obtained using the WTA algorithm are generally noisy, and the results of the CP algorithm have a slight detail loss problem. It can be inferred that the dictionary trained using the SGD algorithm contains richer information about the image base.

## 4. Discussion

**Figure 6:**
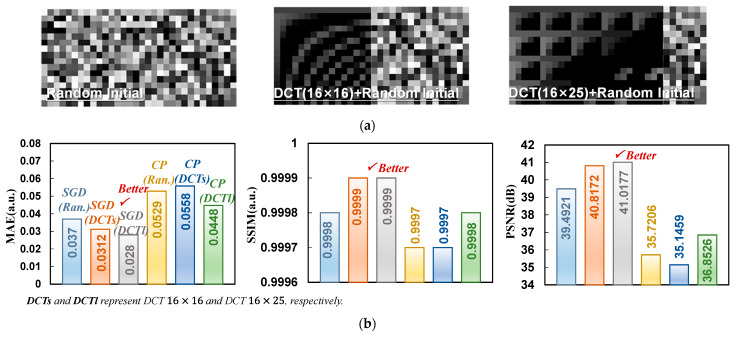
(**a**) Different initial situations. (**b**) The results of CP and SGD algorithms with different initialization conditions are compared in the reconstruction of Lena.

However, a large number of online training leads to multiple programming and erasing of the array and increases power consumption. Since each sparse dictionary can also be treated as a sparse representation of the ‘dictionary of dictionaries’ [30], which means that each element in a sparse dictionary can be treated as a weighted combination of several DCT dictionary elements, we introduce the discrete cosine transform (DCT) matrix to replace the randomly given initial values in the original training method. It should be noted that DCT dictionaries are usually the size of $n^{2}\times n^{2}$, while the dictionaries needed in the dictionary reconstruction process are overcomplete dictionaries, i.e., with the size of $n^{2}\times 2n^{2}$, to ensure good reconstruction results. Thus, the reconstruction effect of ‘Lena’ with different sizes of DCT matrices is investigated firstly to replace the random initial values. From Figure 6, it is concluded that the replacement of the original random dictionaries leads to an enhancement in the quality of the reconstruction results obtained. On the other hand, we can also draw the conclusion that by introducing the DCT matrix to replace the original random initialization matrix, the original good reconstruction effects could be achieved with a reduced number of training epochs, which implies a significant amount of power consumption saving during online training.

For a more comprehensive measurement of the on-chip training results of the three methods, the robustness of the three methods to noise was also investigated due to the presence of current disturbances caused by the device itself and the environment in the on-chip training and sparse coding reconstruction process.

**Figure 7:**
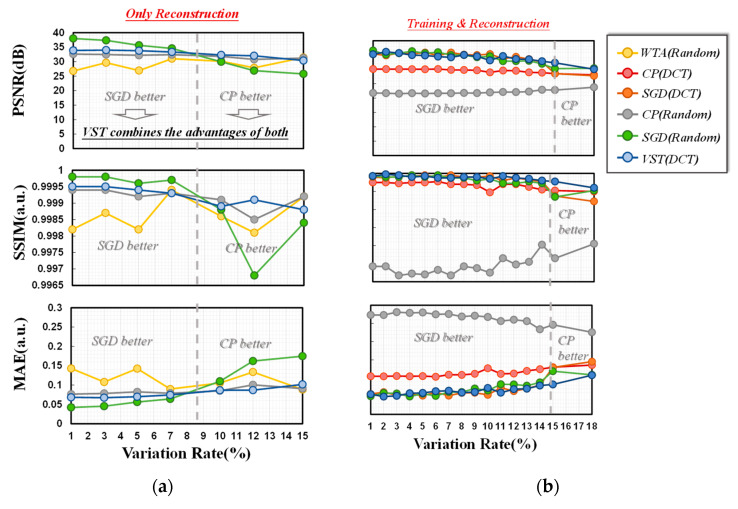
(**a**) Effects of different current variation rates during the reconstruction process on the results are displayed. (**b**) Effects of variations during both the online training and the reconstruction process are taken into consideration.

First, the effect of the noise during on-chip reconstruction on the results is focused. Figure 7a shows that the dictionary trained using the SGD method maintains a good reconstruction when the current disturbance is below a certain level, while the one trained via the CP algorithm is superior when the disturbance reaches a relatively large level since, during each SGD update, only one sample is used. Taking the disturbances during online training into consideration, the final results are shown in Figure 7b. Similar to the case where variation during online training is not taken into account, the SGD method still suffers from reduced robustness in the presence of a high variation level. To overcome this limitation, a variation-sensitive training (VST) method is proposed.

As described in Algorithm 1, the training iteration number of the second training algorithm (CP) should be increased when the current variation is larger. And in the opposite case, the training iteration number of the first algorithm (SGD) should be increased.

**Figure 8:**
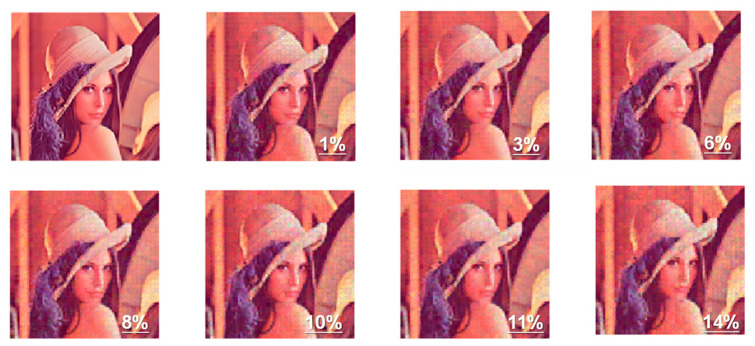
VST-based reconstructions with different variation rates.

To verify the feasibility of this algorithm, the effect of the current variation on the newly proposed algorithm with the optimized initialization was investigated, as shown in Figure 7 (blue line). The reconstructed images suffering different current variation rates are displayed in Figure 8. The reconstruction results maintained a good state regardless of the current variation, with or without taking variation during online training into consideration. Overall, we have demonstrated that flash arrays can be used for sparse coding applications, and the feasibility of the VST algorithm with DCT initialization conditions and optimized mapping method on flash arrays have also been illustrated; enhanced robustness and better reconstruction effects can be achieved as well as less array area.
**Algorithm 1**: The Variation-Sensitive-Training Algorithm**INPUT:** x: a set of sample pics;D: initial dictionary, i.e., threshold voltage;iter1: the iteration number of first training process;iter2: the iteration number of last training process;
iter3: the LCA iteration number;λ,τ: parameters of training;**OUTPUT:** D: trained dictionary;
Initial C=P=0,u=a=0;***#LCA***
for  l=1 to iter1
for  i=1 to iter3$B=D^{T}x;$***#threshold judgment***if u>λ a=u*;* else a=0;end if$x_{r}=Da^{T}$;$H={\left(x-x_{r}\right)}^{T}D$;$du=\frac{1}{\tau }\left(-u+H+a\right)$;u=u+du;end for***#update weight (less noise)***$\Delta \emptyset^{T}=\beta \left(x-D^{T}a\right)⊗a$;for l=iter1+1 to iter2$c^{l}=c^{l-1}+a^{l}a^{lT}$;$p^{l}=p^{l-1}+x^{l}a^{lT}$; ***#update weight (greater noise)***$D_{j}=\frac{1}{\max\left(\|\phi_{j}{\|}_{2},1\right)}\phi_{j}$;$\phi_{j}=\frac{1}{c\left[j,j\right]}\left(p_{j}-Dc_{j}\right)+D_{j}$;end forend for

## 5. Conclusions

Sparse coding holds crucial significance across various domains, ranging from signal compression and denoising in signal processing to extracting essential patterns for improved model generalization in machine learning, even extending to neuroscience. To address the limitations in the exploration of sparse coding applications on flash memory, we present a flash memory-based array designed for sparse coding implementation that enables on-chip training and image reconstruction, demonstrating the ability of flash memory to implement sparse coding. The effects of three dictionary training methods used in the image reconstruction process were investigated, and the performances of these three dictionaries in image reconstruction were analyzed, while a new mapping method was introduced to reduce the array area. In addition, we investigated the effect of device variation on the reconstruction result, and the VST method with a novel initialization condition to implement a color image reconstruction process combining the advantages of both algorithms was proposed. Based on 55 nm technology flash memory, good reconstruction effects were achieved, as well as enhanced robustness to noise.

## Figures and Tables

**Table 1:** The reconstruction effects of different methods on three different images.

Method	*PSNR* (dB)			*SSIM* (a.u.)			*MAE* (a.u.)		
	Lena	Barbara	Kid	Lena	Barbara	Kid	Lena	Barbara	Kid
WTA	27.7296	29.0198	30.5530	0.9991	0.9995	0.9996	0.1323	0.1037	0.0811
CP	32.7659	32.8162	31.4524	0.9995	0.9995	0.9990	0.0747	0.0722	0.0875
SGD	38.4365	40.4555	39.9035	0.9998	1.0000	0.9999	0.0398	0.0291	0.0336

## Data Availability

The data presented in this study are available upon request from the corresponding author. The data are not publicly available due to privacy restrictions.
